# Aging and the detection of moving objects defined by common fate

**DOI:** 10.1038/s41598-022-25456-z

**Published:** 2022-12-02

**Authors:** J. Farley Norman, Maheen Baig, Jiali D. Graham, Jessica L. Lewis

**Affiliations:** 1 Department of Psychological Sciences, Ogden College of Science and Engineering, Western Kentucky University, 1906 College Heights Blvd. #22030, Bowling Green, KY 42101-2030 USA; 2 Center for Applied Science in Health and Aging, Western Kentucky University, Bowling Green, KY 42101-2030 USA; 3 Department of Psychological Sciences, Ogden College of Science and Engineering, Western Kentucky University, Bowling Green, KY 42101-2030 USA; 4 Carol Martin Gatton Academy of Mathematics and Science, Bowling Green, KY USA

**Keywords:** Human behaviour, Perception

## Abstract

Grouping by common fate plays an important role in how human observers perceive environmental objects. In this study, the effect of aging upon the ability to utilize common fate was evaluated. Twenty-two younger and older adults (mean ages were 23.4 and 74.7 years, respectively) participated in two experiments. On any given trial, the participants sequentially viewed two apparent motion sequences and were required to indicate which temporal interval contained a coherently moving dotted line embedded in noisy random background motion. In Experiment 1, the number of dots defining the target was varied, while in Experiment 2, the target interpoint spacing was varied. The younger adults outperformed the older adults by 19.4 percent in Experiment 1 and 50.5 percent in Experiment 2. The older and younger adults were similarly affected by variations in the number of target dots and the target interpoint spacing. The individual older participants’ object detection accuracies were highly correlated with their individual chronological ages, such that the performance of the younger old participants was much higher than that exhibited by the older old. Increases in age systematically affect the ability of older adults to detect and visually perceive objects defined by common fate.

## Introduction

When we look out at the world, we see a world filled with environmental objects. In many natural scenes, stationary objects are effectively camouflaged against their background. When such objects move relative to the background, however, the camouflage is effectively broken^1,2^. About a hundred years ago, Wertheimer^3^ concluded that if any set of stimulus elements (could be texture elements on a visible object surface) share a “common fate” that is different from other stimulus elements (e.g., a particular subset of stimulus elements moves *together* with a particular direction and speed), they will be grouped together into a single phenomenal object that is perceptually segregated from other objects and the background.

A very interesting set of experiments evaluating perceptual grouping by common fate was conducted by Uttal and colleagues^4^. In their experiments, observers viewed two apparent motion sequences on each trial. Pure random motion was shown in one of the two temporal intervals (100 dots, with each dot translating in its own randomly determined direction); 100 moving dots were also shown in the other temporal interval, but in this case, 3 to 5 colinear dots moved together in the same direction against the random motion background. The observers’ task was to determine in which temporal interval (first or second) the colinear dots appeared. Uttal et al. found that both the (1) number of colinear dots and (2) the interpoint spacing between them affected the detectability of the moving dotted line segment.

Another study documenting the importance of common fate was conducted by Lappin, Norman, and Mowafy^5^. In addition to the translation studied by Uttal et al.^4^, Lappin et al. sought to determine whether human observers could successfully detect objects defined by other types of common motion (coherent rotation, expansion/contraction, and shear). On any given trial in Experiment 1, a pattern of eight dots either translated, rotated, expanded/contracted, or underwent a shear transformation. On half of the trials, the same appropriate magnitude and direction of motion was applied to all of the eight stimulus dots; Lappin et al. referred to this as “correlated” or “coherent” motion. For the remaining trials depicting uncorrelated motion, each dot underwent the same transformation (translation, rotation, expansion/contraction, or shear), but a different magnitude and/or direction of the motion was applied to each individual stimulus dot. The observers’ task was to indicate for each stimulus whether it depicted correlated or uncorrelated motion. The results indicated that the observers were equally sensitive at detecting the coherent motion of the dotted patterns regardless of the particular type of transformation. As can be seen by the review of the literature so far, human adults are very good at detecting different forms of common fate and using it to successfully perceive moving objects.

What about older adults? Can they utilize common fate as well as younger adults when perceiving environmental objects? Given that older adults possess difficulty in perceiving both components of motion (direction and speed^6–14^), their abilities to detect common fate may be reduced as well. In one of the only existing studies of aging and common fate to date, Norman, Sanders, Shapiro, and Peterson^15^ presented observers with stimulus displays containing 4,900 rotating line segments. As in the earlier study by Julesz and Hesse^16^, a subset of these line segments (within a rectangular region) rotated at a different speed. The observers’ task was to judge whether this motion-defined rectangle was horizontally or vertically oriented. Norman et al. found that while older adults could reliably discriminate the shape of the rectangles defined by common fate, their discrimination performance was nevertheless much lower than that of the younger participants (e.g., when compared to the older adults in comparable conditions, the younger adults’ shape discrimination accuracies, d’ values, were 71.4 percent higher).

The primary goal of the current study was to extend the small literature concerning aging and the perception of common fate by adopting the methodology used by Uttal et al.^4^. In the prior experiment of Norman et al.^15^, the younger and older observers were required to discriminate the shape of an object defined by common fate. In the experiments performed by Uttal et al., the observers were only required to detect the *presence* of a moving object defined by common fate. Perhaps the large and negative effect of age obtained by Norman et al.^15^ will be reduced or eliminated due to the simpler nature of the required task (i.e., simple detection of objects, instead of judging their shape). Even if older adults perform well at simple detection of objects defined by common fate, will they be differentially affected by reductions in the number of dots defining the stimulus objects or by increases in interpoint spacing? The specific purpose of the current two experiments is to answer such questions. Given that the “segregation of objects from their backgrounds is one of vision’s most important tasks” (p. 2)^17^ and that there are other ways besides motion by which segregation is achieved (see Regan^18^), any finding of an age-related deficit in the current study may reflect a more general decline in basic perceptual functioning.

## Experiment 1

### Method

#### Apparatus

The apparent motion sequences were generated and presented by an Apple M1 iMac computer. The same computer was used to record the participants’ responses.

#### Experimental stimuli

**Figure 1:**
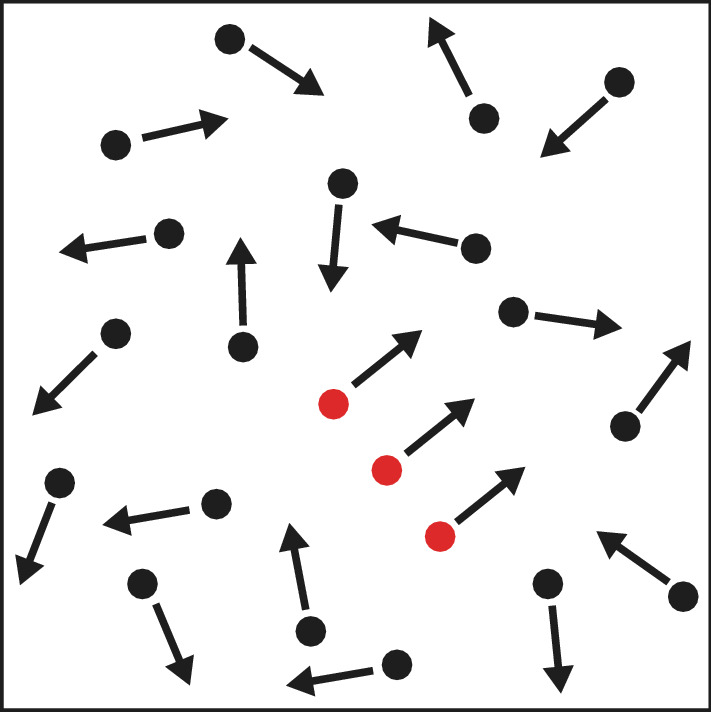
A diagram illustrating the basic common fate stimulus employed in the current experiments. Most of the stimulus points moved (translated) in randomly determined directions (indicated by the vectors). A subset of three to five points, however, moved together with the same direction and speed; note that in this figure three points (towards the lower right) are moving upwards and to the right (it is important to note that in the actual stimulus displays, all points were yellow presented against a black background). This common motion causes the participant to see a coherent object moving against a “noisy” motion background.

The stimulus displays were essentially identical to those used by Uttal and colleagues^4^. Two apparent motion sequences were generated and shown on each trial; each apparent motion sequence consisted of 120 frames updated at a refresh rate of 60 Hz (the duration of each apparent motion sequence was therefore 2.0 s). In one of the two temporal intervals (randomly determined on each trial), each of the 100 moving points (yellow points presented against a black background) translated along in its own randomly determined direction. If a point reached the edge of the stimulus window, it reappeared at the opposite side and continued to move in its original direction. As in the experiments by Uttal et al., the lifetime of each point was limited (to 80 frames). When an individual point had survived for 80 frames, it then “died”, and a new point was inserted at a randomly determined position within the stimulus pattern. This new point then moved along its own randomly determined direction for either the duration of its lifetime or the remainder of the temporal interval, whichever came first. At the beginning of an apparent motion sequence, the phase of each stimulus point within its total lifetime was randomly determined so that the individual stimulus points appeared and disappeared at different times throughout the temporal interval. Notice that within this temporal interval, there was no coherent motion of any object, just the “noisy” motion of 100 randomly moving points. Most of the stimulus points in the other temporal interval behaved in the exact same manner—95 to 97 points moved in the same manner as previously described. However, in this apparent motion sequence 3 to 5 stimulus points were constrained to form a dotted line segment (randomly determined orientation) where the interpoint spacing was 27 min arc (0.45 deg visual angle). These 3 to 5 points moved together (see Fig. 1) in a direction perpendicular to the dotted line’s orientation for a duration of 80 frames (1.33 s). As in the experiments by Uttal et al., the points forming the dotted line segment (i.e., the target), did not appear at the beginning of the relevant temporal interval, but at a random delay (subject to the provision that the target dotted line segment must be presented for the required 80 frames). All stimulus points moved with a speed of 2.6 deg/sec.

The two temporal intervals of each trial (one containing only “noise” and one containing a moving target embedded in a noisy background) were presented successively within a 600 × 600 pixel window that subtended (in both width and height) 6.94 deg visual angle. The viewing distance was 114.6 cm.

#### Procedure

Each participant made judgments for a total of 120 trials (40 trials for targets defined by 3 points, 40 trials for targets defined by 4 points, and 40 trials for targets defined by 5 points). On each trial, the choice of condition (i.e., the number of points defining the target dotted line segment) was random. Following each trial, the participant indicated which temporal interval (first or second) contained the target (i.e., the moving dotted line segment). No feedback was given during the block of 120 trials. To ensure that all participants understood the stimulus and task, they participated in a block of 40 trials prior to beginning the experiment (the target dotted line segment for these trials contained 4 points). To help explain the task, the points forming the dotted line segment were red, while the background points were yellow. All participants were able to see the moving target line segment and indicate in which temporal interval it appeared with an accuracy of 98.2 percent correct (it is important to remember that this color difference, between target and background points, *only* occurred during these 40 familiarization trials and did not exist during the actual experiment).

#### Participants

If an effect of age exists for the current task that is comparable to that obtained in our recent study of perceiving object shape defined by common fate^15^, a power analysis revealed (for a power of 0.85) that we would need a sample of 11 older adults and 11 younger adults to detect it. In the current investigation, we therefore recruited a total of 22 naive participants (11 younger adults and 11 older adults). The mean ages of the younger and older participants were 23.4 years (ages ranged from 20 to 29 years, sd = 3.1) and 74.7 years (ages ranged from 67 to 83 years, sd = 3.9), respectively. One potential older participant (age was 80 years) was unable to perform the task at better than chance levels during the familiarization trials and therefore did not participate in the experiment. The participants had good visual acuity: the acuity of the younger and older observers measured at 100 cm was − 0.14 and − 0.06 LogMAR (log minimum angle of resolution) for the younger and older participants, respectively (zero LogMAR represents normal visual acuity, while negative and positive values represent better than and worse than normal acuity, respectively). The study was approved by the Institutional Review Board of Western Kentucky University, and each participant signed an informed consent document prior to testing. Our research was carried out in accordance with the Code of Ethics of the World Medical Association (Declaration of Helsinki).

## Results

**Figure 2:**
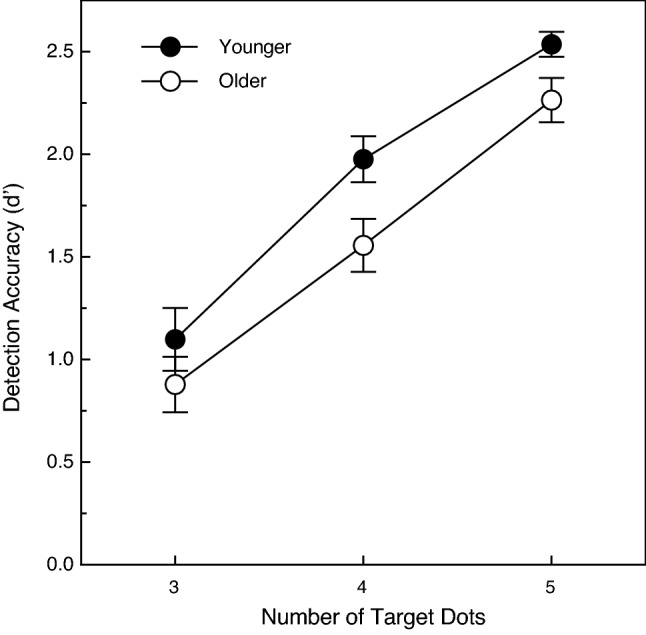
The younger and older participants' object detection accuracies (d’ values) plotted as a function of the number of target dots. The younger and older participants' results are indicated by filled and open circles, respectively. The error bars indicate ± 1 SE. One can see that as the number of target dots was increased, the participants’ detection accuracy increased at essentially the same rate for younger and older adults.

**Figure 3:**
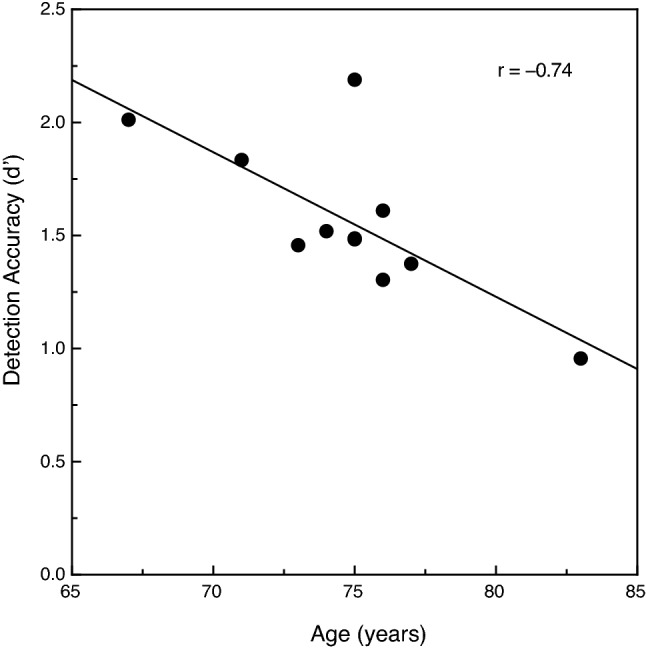
The individual older participants' overall object detection accuracies (d’ values) plotted as a function of chronological age. The solid line indicates the best-fitting linear regression. It is clear that the older participants’ detection accuracy is best for the younger old participants and deteriorates with increasing age.

The overall results are shown in Fig. 2. We evaluated the participants’ sensitivity to the object (defined by common fate) in terms of d’, the perceptual sensitivity measure of signal detection theory^19^. The participants’ performance increased as the number of points defining the objects increased (F (2, 40) = 104.5, *p* < 0.000001; η^2^_p_ = 0.84). The participants’ d’ values for the objects defined by 5 dots was 2.4 times higher than for the objects defined by 3 dots. The younger adults’ performance was 19.4 percent higher than that of the older adults; this age-related difference in performance was significant (F (1, 20) = 5.8, *p* = 0.026; η^2^_p_ = 0.22). The effect of the variations in the number of target points was similar in magnitude for both the younger and older adults; this was reflected by the absence of an age x number of target points interaction (F (2, 40) = 0.6, *p* = 0.58, η^2^_p_ = 0.03). Given that previous investigations involving motion-related tasks^10,20^ found considerable variations in performance within older groups of participants such that the younger old performed better than the older old, we decided to plot our individual older participants’ object detection performance as a function of their chronological ages. These results are shown in Fig. 3. It is clear that the older participants’ overall detection accuracies declined substantially as their individual ages increased (Pearson r = − 0.74, *p* = 0.01, 2-tailed). The d’ value for the youngest older adult (67 years) was more than twice that obtained for the oldest older adult (83 years).

## Experiment 2

In Experiment 1, we varied the number of stimulus points defining the object formed by common fate. In the second experiment, we kept the number of points constant (always 4) but varied the spacing between the target’s constituent points. Uttal et al.^4^ found that variations in target point spacing significantly affected the detection performance of younger adults; will such variations in interpoint spacing affect older adults differentially, or will their performance be similarly affected? The purpose of Experiment 2 was to answer this question.

### Method

#### Apparatus

The same computer that was used in Experiment 1 was also used to generate and present the experimental stimuli in this experiment.

#### Experimental stimuli

The experimental stimuli for this experiment were almost identical to those used in Experiment 1. In this experiment, the target objects (dotted lines) were always defined by four points, which were separated by either 25, 32, or 39 min arc. All other aspects of the stimuli and apparent motion sequences were identical to those used in Experiment 1.

#### Procedure

As in Experiment 1, 40 trials were completed for each of the three experimental conditions (target interpoint spacings of 25, 32, & 39 min arc). As a result, each participant made a total of 120 judgments. The order of the three magnitudes of interpoint spacing across trials was randomly determined for each participant. The participants’ task was the same as in Experiment 1, to indicate on each trial whether the first or second temporal interval contained the moving target object defined by common fate (i.e., common motion).

#### Participants

The same 22 younger and older adults who had participated in Experiment 1 also participated in this experiment. The study was approved by the Institutional Review Board of Western Kentucky University, and each participant signed an informed consent document prior to testing. Our research was carried out in accordance with the Code of Ethics of the World Medical Association (Declaration of Helsinki).

## Results

**Figure 4:**
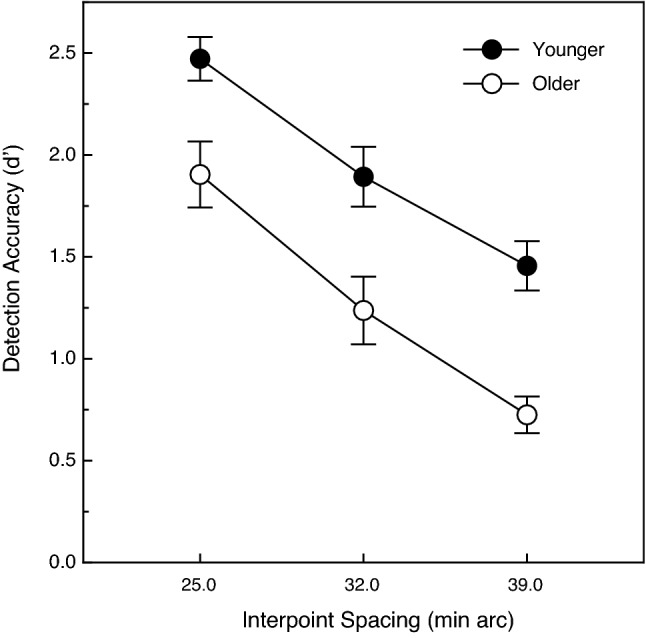
The younger and older participants' object detection accuracies (d’ values) plotted as a function of the target interpoint spacing. The younger and older participants' results are indicated by filled and open circles, respectively. The error bars indicate ± 1 SE. One can see that as the target interpoint spacing increased, the participants’ detection accuracy decreased at essentially the same rate for younger and older adults.

**Figure 5:**
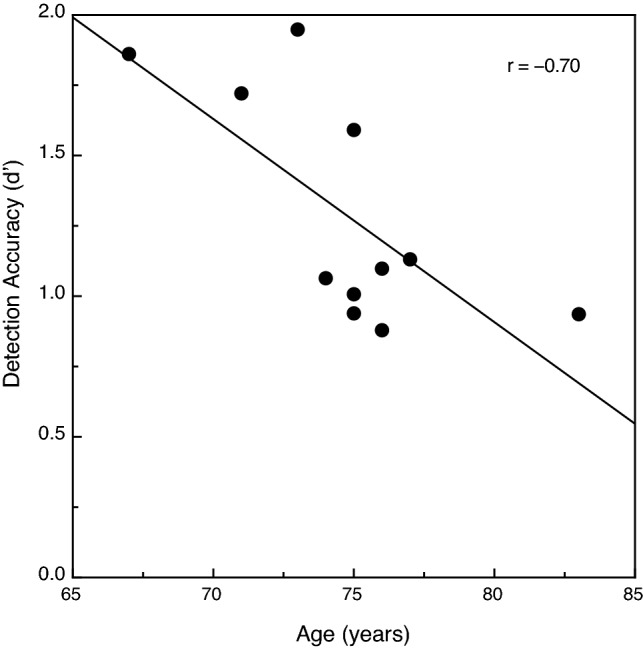
The individual older participants' overall object detection accuracies (d’ values) plotted as a function of chronological age. The solid line indicates the best-fitting linear regression. The older participants’ detection accuracy is best for the younger old participants and deteriorates with increasing age.

The overall results for this experiment are shown in Fig. 4. The participants’ performance decreased as the target interpoint spacing increased (F (2, 40) = 67.1, *p* < 0.000001; η^2^_p_ = 0.77). The participants’ d’ values for the objects with 25 min arc interpoint spacing was 2.0 times higher than for the objects with 39 min arc interpoint spacing. The younger adults’ performance was 50.5 percent higher than that of the older adults; this age-related difference in performance was significant (F (1, 20) = 17.3, *p* < 0.001; η^2^_p_ = 0.46). The effect of the variations in target interpoint spacing was similar in magnitude for both the younger and older adults; this was reflected by the absence of an age x target interpoint spacing interaction (F (2, 40) = 0.37, *p* = 0.69; η^2^_p_ = 0.02). Once again, we plotted our individual older participants’ object detection performance as a function of their chronological ages. These results are shown in Fig. 5. Just as in Experiment 1 (see Fig. 3), the older participants’ overall detection accuracies declined significantly as their individual ages increased (Pearson r = − 0.70, *p* = 0.017, 2-tailed). The performance (i.e., d’ value) for the three youngest older adults, for example, was nearly twice (1.97 times higher) that exhibited by the oldest older adult (83 years).

**Figure 6:**
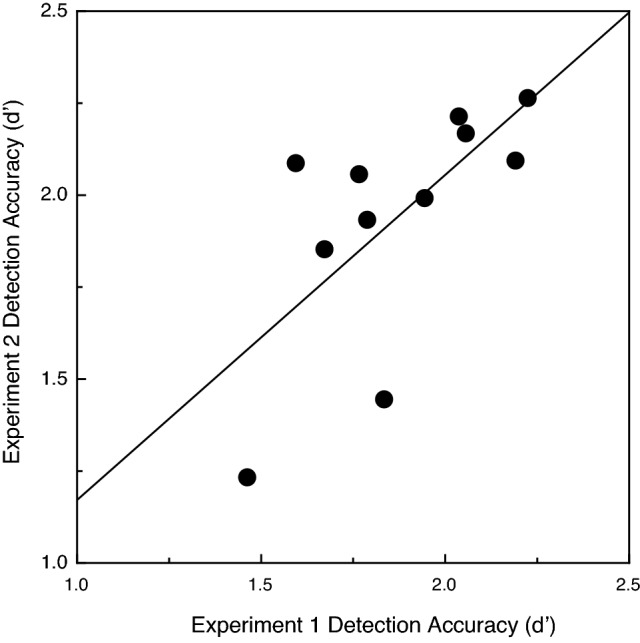
The individual younger participants' overall object detection accuracies (d’ values) in Experiment 2 are plotted as a function of their analogous performance in Experiment 1. The solid line indicates the best-fitting linear regression.

**Figure 7:**
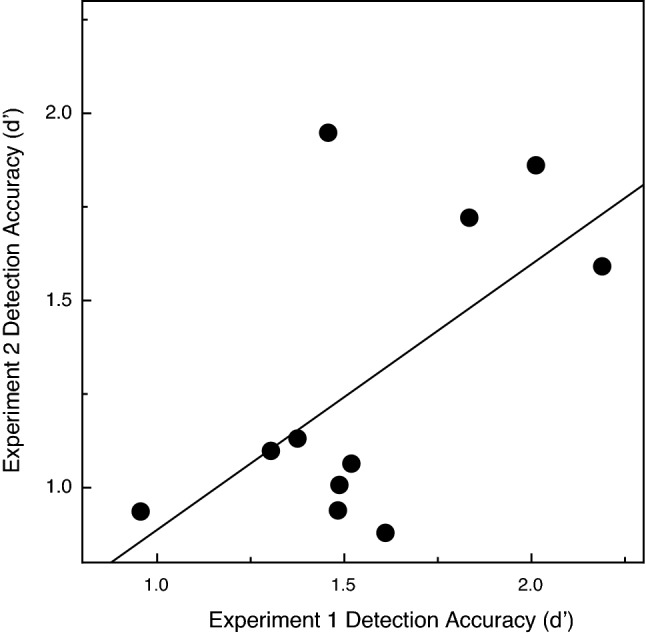
The individual older participants' overall object detection accuracies (d’ values) in Experiment 2 are plotted as a function of their analogous performance in Experiment 1. The solid line indicates the best-fitting linear regression.

Because the same 22 observers participated in both experiments, we decided to plot their object detection performance obtained in Experiment 1 with that of the current Experiment. If the individual participants are consistent in their ability to utilize and detect common fate, one would logically expect that participants whose performance was higher in Experiment 1 would also perform well in Experiment 2, and that those with less sensitivity to the motion-defined object in Experiment 1 would be similarly less sensitive in Experiment 2 (i.e., one would expect a positive correlation between performance obtained in the two experiments). Indeed, this was the case. Figures 6 and 7 plot the younger and older participants’ d’ values obtained in Experiment 2 as a function of the analogous performance obtained in Experiment 1. The correlation coefficients (Pearson r) were significant for both the younger (r = 0.67, *p* = 0.012, 1-tailed) and older adults (r = 0.6, *p* = 0.026, 1-tailed).

## General discussion

Using stimulus displays very similar to those of the current investigation, Uttal and colleagues^4^ found that their observers’ object detection performance improved with increasing numbers of target points and decreases in target interpoint spacing (see their Figs. 1 and 2). Our results (see current Figs. 2 and 4) replicate their findings and additionally demonstrate that the ability of older adults to visually perceive objects defined by common fate is qualitatively similar to that of younger adults. For example, the judgments of both younger and older adults are affected similarly by variations in the number of target points and target interpoint spacing. Nevertheless, it is also true that our older adults’ perceptual sensitivity to the motion of objects defined by common fate was significantly lower (see Figs. 2 and 4). Given the obvious importance of visually detecting moving objects in everyday life, it is perhaps surprising that no one has ever published an article evaluating aging and common fate perception using methods similar to those employed in the current study.

The results of the current experiments not only document an overall adverse effect of age upon the visual ability to detect objects defined by common fate, they also indicate that this ability declines as a linear function of chronological age (at least over the range of 67 to 83 years of age). In Experiment 1 (see Fig. 3), the older participants’ d’ values (perceptual sensitivities) declined by 0.64 per decade. The corresponding decrease in d’ values for the older adults in Experiment 2 was 0.72 per decade. Fjell et al.^21^ measured the rate of brain atrophy in 142 healthy older adults across the entire cerebral cortex and for many important subcortical structures. They found significant (up to 0.5 percent loss of grey matter per year) brain atrophy for most areas within the parietal and temporal lobes, as well as in the thalamus (see Table 2 of Fjell et al., especially the atrophy rates across 2 years). It is important at this point to remember that much of the parietal and temporal lobes of the cerebral cortex constitute extrastriate visual cortex, which is responsible for a wide variety of visual functions (also remember that the thalamus contains critical nuclei for vision, such as the lateral geniculate nucleus and pulvinar). Given that the cortical and subcortical brain atrophy documented by Fjell et al. compounds over time (imagine a loss of 0.5 percent of a particular brain area or structure occurring each and every year as one ages), the cumulative effects will necessarily be large. Because of this continuing and progressive atrophy in visual extrastriate cortex, it is perhaps surprising that our older participants performed as well as they did in the current experiments (and that their d’ values only decreased by 0.64 to 0.72 per decade).

The results of the current experiments are generally consistent with a large body of research (reviewed by Billino & Pilz^22^) that has documented adverse effects of increased age for many other different tasks involving motion. Such tasks would include (1) judging the speed of motion^6–8^, (2) judging the direction of motion^9–14^, and (3) judging the 3-dimensional shape of objects from motion^20,23–25^. The study by Tadin et al.^17^ is particularly relevant. On any given trial, their younger and older participants judged the orientation of a small motion-defined ellipse (e.g., if the random texture pattern within the elliptical region moved down, the texture outside the ellipse moved up). Their older adults needed a much longer stimulus duration (see Fig. 3d of Tadin et al.) in order to perform this object orientation discrimination task at a threshold level. At this point, it is important to consider together the current results and those of Tadin et al. In both studies, participants made judgments about a motion-defined target embedded against a background of other stimulus elements that moved differently. In 1985, Allman, Miezin, & McGuinness^26^ demonstrated that the activity of neurons (in cortical area MT) detecting the motion of a centrally moving pattern of dots was modulated by the motion of other “background” dots moving within large surrounding regions (see Fig. 6, Fig. 7, Fig. 8 of^26^). Allman et al. concluded (p. 123) that the classical receptive fields “and their surrounds provide mechanisms for local–global comparisons embedded in visuotopic matrices that may serve as the basis for many functions in vision such as … figure-ground discrimination”. It is therefore almost certainly the case that motion-sensitive neurons with center/surround receptive fields are critical for the tasks used both in the current study and that of Tadin et al.^17^. A 2005 study by Betts, Taylor, Sekuler, and Bennett^27^ demonstrated that the inhibitory effects of surrounding motion were reduced by aging—this interestingly allows older adults to better discriminate the direction of high-contrast common motion that occurs across large areas of the visual field. While age-related deteriorations in surround suppression produce improvements in some aspects of motion perception^27^, the current results and those of Tadin et al.^17^ now demonstrate that this same age-related reduction in surround suppression leads to a reduced ability to segregate figure from ground (e.g., see our Fig. 2, Fig. 3, Fig. 4, Fig5).

The task used in the current experiments required perceptual grouping across space (to perceive and thus detect the dotted line defined by common fate). Other studies have also found age-related deficits for tasks involving spatial integration^28–31^, even when the stimulus patterns are stationary and do not include motion^29–31^. As examples, consider the studies by Roudaia, Farber, Bennett, and Sekuler^30^ and Del Viva and Agostini^31^. In any given trial of Experiment 2 of Roudaia et al., participants judged which of two intervals contained a C-shaped contour defined by separated Gabor patches. In the study by Del Viva and Agostini, participants selected which quadrant of the stimulus display (upper left, upper right, lower right, lower left) contained a circularly-shaped target composed of separated Gabor patches. In both experiments, the participants were required to detect a contour (defined by spatially separated Gabor patches) that was embedded in a background of “noise” (other Gabor patches). It is probably important to note that in our current study, our participants were also required to detect a coherent object embedded against a background of noise. In the study by Roudaia et al., the older participants needed longer stimulus durations to detect the target objects, while in the experiment by Del Viva and Agostini, their older participants could detect the target object contours successfully, but only with fewer background noise elements.

Given the adverse effects of age obtained for a wide variety of motion-related tasks and tasks requiring spatial integration, it is very interesting that aging is not associated with a deterioration in performance for either shape discrimination tasks performed under full-cue conditions^32^ or for tasks involving the visual discrimination or estimation of environmental distances^33–36^. It is certainly true that the effects of age upon visual perception are not uniform, but depend greatly upon the particular task participants are asked to perform. In their article, Billino et al.^12^ conclude (p. 1254) by saying “We suggest that differential age effects on the perception of specific motion types might indicate that specialized neuronal processing mechanisms differ in their vulnerability to physiological changes during aging”. Our current and previous results^6,9,15,20,23–25,32,34–36^ reinforce the conclusions of Billino et al. and extend them to a wider domain of perceptual functioning.

## Conclusion

While older adults can successfully perceive objects defined by common fate, their detection performance is nevertheless significantly lower than that exhibited by younger adults. In addition, older adults’ ability to perceive motion-defined objects deteriorates as a function of chronological age, such that the performance of older old adults is significantly reduced relative to younger old adults.

## Data Availability

The datasets generated during and/or analyzed during the current study are available from the corresponding author on reasonable request.
